# Complete Genome Sequence of a Newcastle Disease Virus Isolate from an Outbreak in Northern India

**DOI:** 10.1128/genomeA.00342-14

**Published:** 2014-04-24

**Authors:** Sudhir Morla, Ashok Kumar Tiwari, Vinay Joshi, Sachin Kumar

**Affiliations:** a Department of Biotechnology, Indian Institute of Technology Guwahati, Guwahati, Assam, India; b Standardization Division, Indian Veterinary Research Institute, Izatnagar, Bareilly, Uttar Pradesh, India

## Abstract

The complete genome sequence was determined for a Newcastle disease virus strain from vaccinated chicken farms in India during outbreaks in 2010. The genome is 15,192 nucleotides (nt) in length and is classified as genotype VII in class II. Compared to the available vaccine strains, the Indian strain contains a previously described 6-nt insertion in the untranslated region of the nucleoprotein gene.

## GENOME ANNOUNCEMENT

Newcastle disease (ND) is a highly infectious viral disease of avian species. ND is economically significant because of the huge mortality and morbidity associated with it. Newcastle disease virus (NDV) belongs to the genus *Avulavirus* in the family *Paramyxoviridae*. The genome of NDV is a nonsegmented, negative-sense, single-stranded RNA (1). The NDV strains isolated from different parts of the world fall into three genome size groups: 15,186 nucleotides (nt) long in the isolates before 1960, 15,192 nt long in the isolates discovered in China, and 15,198 nt long in the avirulent strain from Germany (2). NDV is classified into classes I and II based on genetic analysis. Class I is composed of avirulent strains isolated from wild birds, whereas class II is composed of both virulent and avirulent strains isolated from wild and domestic birds (3).

In India, there have been continuous outbreaks of ND, leading to serious losses to domestic poultry industries, in spite of vaccination (4). In 2010, ND outbreaks affected chicken farms located in the Bareilly province in the state of Uttar Pradesh in India. ND outbreaks occurred in commercial vaccinated chickens, causing up to 35% to 40% mortality. Tissue samples from the trachea and lungs are collected under aseptic conditions from dead and sick birds exhibiting signs of the disease. The virus was isolated by inoculating the tissue homogenates into 9-day-old embryonated chicken eggs. One of the isolates, namely, NDV/Chicken/Bareilly/01/10, was purified, and the genome sequence was determined by reverse transcription (RT)-PCR using overlapped consensus primers and direct sequencing (5). Rapid amplification of cDNA ends (RACE) was used to determine the 3′ and 5′ ends of the viral genome (6).

The complete genome of NDV/Chicken/Bareilly/01/10 is 15,192 nt in length. The amino acid sequence identities of the fusion (F) and hemagglutinin-neuraminidase (HN) proteins between NDV strain NDV/Chicken/Bareilly/01/10 and the normal vaccine strain LaSota are 89% and 86%, respectively. The findings suggest that the circulating strains are substantially distinct from the vaccine strain in use. The antigenic differences between the vaccine and circulating NDV strains contributed to poor vaccine protection and a subsequent outbreak.

The sequence of the F protein cleavage site is a major determinant of NDV pathogenicity. The strain NDV/Chicken/Bareilly/01/10 has a virulent pathotype, with ^112^RRQKR^116^ at the C terminus of the F2 protein and F at residue 117 (7). Phylogenetic analysis of the F gene by MEGA 4.0 suggests that the present isolate belongs to genotype VII of class II (8).

### Nucleotide sequence accession number.

The complete genome sequence of NDV/Chicken/Bareilly/01/10 is deposited in GenBank under the accession no. KJ577585.
